# Monitoring of Unfractionated Heparin in Severe COVID-19: An Observational Study of Patients on CRRT and ECMO

**DOI:** 10.1055/s-0040-1719083

**Published:** 2020-11-19

**Authors:** Alexander S. Streng, Thijs S.R. Delnoij, Mark M.G. Mulder, Jan Willem E.M. Sels, Rick J.H. Wetzels, Paul W.M. Verhezen, Renske H. Olie, Jeroen P. Kooman, Sander M.J. van Kuijk, Lloyd Brandts, Hugo ten Cate, Roberto Lorusso, Iwan C.C. van der Horst, Bas C.T. van Bussel, Yvonne M.C. Henskens

**Affiliations:** 1 Central Diagnostic Laboratory, Maastricht University Medical Centre, Maastricht, the Netherlands; 2 Intensive Care Unit, Maastricht University Medical Centre, Maastricht, the Netherlands; 3 Cardiovascular Centre, Maastricht University Medical Centre, Maastricht, the Netherlands; 4 Department of Vascular Medicine, Maastricht University Medical Centre, Maastricht, the Netherlands; 5 Cardiovascular Research Institute Maastricht, Maastricht University, Maastricht, the Netherlands; 6 Department of Clinical Nephrology, Maastricht University Medical Centre, Maastricht, the Netherlands; 7 Clinical Epidemiology and Medical Technology Assessment, Maastricht University Medical Centre, Maastricht, the Netherlands; 8 Care and Public Health Research Institute, Maastricht University Medical Centre, Maastricht, the Netherlands; 9 Department of Cardiothoracic Surgery, Heart and Vascular Centre, Maastricht University Medical Centre, Maastricht, the Netherlands

**Keywords:** COVID-19, heparin resistance, heparin therapeutic range, aPTT, anti-Xa

## Abstract

**Objective** Severe cases of coronavirus disease 2019 (COVID-19) can require continuous renal replacement therapy (CRRT) and/or extracorporeal membrane oxygenation (ECMO). Unfractionated heparin (UFH) to prevent circuit clotting is mandatory but monitoring is complicated by (pseudo)-heparin resistance. In this observational study, we compared two different activated partial thromboplastin time (aPTT) assays and a chromogenic anti-Xa assay in COVID-19 patients on CRRT or ECMO in relation to their UFH dosages and acute phase reactants.

**Materials and Methods** The aPTT (optical [aPTT-CS] and/or mechanical [aPTT-STA] clot detection methods were used), anti-Xa, factor VIII (FVIII), antithrombin III (ATIII), and fibrinogen were measured in 342 samples from 7 COVID-19 patients on CRRT or ECMO during their UFH treatment. Dosage of UFH was primarily based on the aPTT-CS with a heparin therapeutic range (HTR) of 50–80s. Associations between different variables were made using linear regression and Bland–Altman analysis.

**Results** Dosage of UFH was above 35,000IU/24 hours in all patients. aPTT-CS and aPTT-STA were predominantly within the HTR. Anti-Xa was predominantly above the HTR (0.3–0.7 IU/mL) and ATIII concentration was >70% for all patients; mean FVIII and fibrinogen were 606% and 7.5 g/L, respectively. aPTT-CS correlated with aPTT-STA ( *r* ^2^ = 0.68) with a bias of 39.3%. Correlation between aPTT and anti-Xa was better for aPTT-CS (0.78 ≤ *r* ^2^ ≤ 0.94) than for aPTT-STA (0.34 ≤ *r* ^2^ ≤ 0.81). There was no general correlation between the aPTT-CS and ATIII, FVIII, fibrinogen, thrombocytes, C-reactive protein, or ferritin.

**Conclusion** All included COVID-19 patients on CRRT or ECMO conformed to the definition of heparin resistance. A patient-specific association was found between aPTT and anti-Xa. This association could not be explained by FVIII or fibrinogen.

## Introduction

In patients with severe cases of coronavirus disease 2019 (COVID-19) admitted to the intensive care unit (ICU), extracorporeal devices such as extracorporeal membrane oxygenation (ECMO) and continuous renal replacement therapy (CRRT) may be temporarily required to support organ function. 1 2 3 Use of ECMO and CRRT results in excessive stimulation of the coagulation system due to blood contact with a variety of nonphysiological surfaces, potentially leading to gradual or sudden thrombosis and the accumulation of thrombi throughout the extracorporeal system, 4 which can result in interruption of such supportive treatments. Therefore, adequate anticoagulation is required during these procedures, 5 6 for which unfractionated heparin (UFH) is commonly used. Furthermore, a prothrombotic condition, in association with a demonstrated hyper-inflammatory state, has been demonstrated in COVID-19 patients, thereby prompting the adoption of higher anticoagulation treatment. 7 8

Monitoring of adequate UFH dosage is essential to maintain the delicate balance between bleeding and thrombosis. The activated partial thromboplastin time (aPTT) reflects the intrinsic coagulation pathway and is the most commonly used test to monitor UFH. 9 10 Activated clotting time and anti-Xa activity tests are also performed. 10 When using the anti-Xa test, practical guidelines suggest a heparin therapeutic range (HTR) of 0.3 to 0.7 IU/mL. 11 12 13 However, those same guidelines are less specific about which HTR to use when monitoring UFH with the aPTT. An aPTT ratio of 1.5 to 2.5 (aPTT of the patient/aPTT of pooled reference plasma) was suggested in a 1972 study and gained wide acceptance. 14 However, other authors have suggested that an aPTT ratio of 2.0 to 3.5 may be more suitable. 15 Because large differences exist between different reagents and platforms, each laboratory should ideally determine the aPTT target range based on a corresponding anti-Xa activity of 0.3 to 0.7 IU/mL. 16 17 In our laboratory, UFH is monitored using the aPTT with a HTR of 50 to 80 s, based on an aPTT ratio of 1.5 to 2.5. We utilize two different instruments for the aPTT: the Sysmex CS2100i (optical clot detection and the default instrument) and the Stago STA-R Max 2 (mechanical clot detection).

Another frequently encountered challenge in heparin monitoring is heparin resistance. Heparin resistance can be defined as the requirement of more than 35,000 units of UFH in 24 hours to reach therapeutic aPTT levels and is classically caused by antithrombin III (ATIII) deficiency. 18 ATIII-independent forms of heparin resistance (also called “apparent,” or “pseudo” heparin resistance) occur as well, possibly caused by high concentrations of FVIII, fibrinogen, or platelets. 19 20 21 Apparent heparin resistance is assumed to be an in vitro effect only, but the mechanisms and clinical meaning of this phenomenon are not fully discerned and may not apply to COVID-19 patients. Nevertheless, international guidelines recommend the use of anti-Xa testing with a HTR of 0.3 to 0.7 IU/mL instead of the aPTT to manage patients with heparin resistance. 11 12

COVID-19 coagulopathy is characterized by high FVIII and fibrinogen concentrations and predominantly normal ATIII levels. 22 During the COVID-19 epidemic, we were confronted with several cases in which the usually effective dose of UFH was insufficient to reach the HTR (defined as an aPTT of 50–80 seconds in our hospital). This prompted us to begin a longitudinal assessment of the aPTT, FVIII, fibrinogen, ATIII, and anti-Xa activity in seven patients diagnosed with COVID-19 on CRRT or ECMO and treated with UFH. The clinical dilemma is that neither the aPTT nor the anti-Xa assays have been assessed for their validity to monitor UFH in the setting of profound thrombo-inflammation such as in COVID-19, where heparin resistance is rather frequently observed. The aim of this explorative study was to compare a mechanical and optical aPTT assay with one another and with the anti-Xa to gain more insight in the causes of heparin resistance and in monitoring strategies for UFH in COVID-19 patients on ECMO or CRRT.

## Materials and Methods

### Patients and Study Design

The Maastricht Intensive Care COVID (MaastrICCht) cohort is a prospective cohort study that is conducted in patients admitted to the ICU of the Maastricht University Medical Center (Maastricht UMC + ). 23 It included 81 patients who had to be intubated and mechanically ventilated and needed to have signs and symptoms of a viral infection including a polymerase chain reaction positive for severe acute respiratory syndrome coronavirus 2 (SARS-CoV-2) and/or a chest CT scan scored positive based on a CORADS-score of 4 to 5 by a radiologist. 24 Patients were admitted via our emergency department, via non-ICU wards and by transportation from other ICUs either for tertiary care referral, such as ECMO, or due to lack of bed availability in the regional hospitals.

As we observed clinically high UFH dosages with low corresponding aPTT result in mechanically ventilated patients with extracorporeal support, we conducted a comprehensive longitudinal study on coagulation using a subcohort of seven consecutive patients receiving therapeutic dosages of UFH for either CRRT or ECMO within a study period of 1 month. ECMO consisted of venovenous extracorporeal membrane oxygenation (VV-ECMO) with the PLS and Cardiohelp HLS system (Maquet Cardiopulmonary, Rastatt, Germany). Indications for VV-ECMO support were a P/F-ratio < 80 mm Hg despite prone positioning and high PEEP (>15 cmH _2_ O), ventilator support <6 days, age <70 years, mono-organ failure, and no severe comorbidities. CRRT was initiated in case of acute kidney insufficiency (AKI) KDIGO stage 3 and consisted of continuous venovenous hemodiafiltration (CVVHD) using the multiFiltrate system (Fresenius Medical Care, Bad Homburg, Germany).

The local institutional review board (Medisch Ethische Toetsingscomissie (METc) 2020–1565/ 300523) of the Maastricht UMC+ waived consent and approved the MaastrICCHt cohort study, in accordance with the Declaration of Helsinki. For the present subcohort, patient families were contacted and provided oral consent.

### Blood Collection, Preparation, and Storage

Atrial blood was routinely collected using 4.0 mL BD (Becton Dickinson, Plymouth, United Kingdom) 7.2 mg K _2_ EDTA, 2.7 mL BD 3.2% citrate, and 5.0 mL BD serum Vacutainer vacuum tubes. Platelet measurements were performed within 2 hours of blood collection. Platelet free plasma (PFP) was obtained by centrifugation of citrated blood at 2000 *g* for 10 minutes and subsequently at 10 000 *g* for 10 minutes at 18°C. PFP was prepared and frozen (-80°C) within 4 hours of blood collection. Routine hematology tests (aPTT, fibrinogen, D-dimers, ATIII) were performed within 2 hours of blood collection. aPTT measurements on the STA-R Max2 intended for assay comparison, as well as Anti-Xa and FVIII were performed in stored PFP (–80°C), thawed for 10 minutes at 37°C.

### Laboratory Measurements

The aPTT (Dade Actin FSL; Siemens, Marburg, Germany), PT (Dade Innovin; Siemens), fibrinogen level (Clauss method, Dade Thrombin Reagent; Siemens), FVIII activity (Dade Actine FS and FVIII deficient plasma; Siemens), D-dimer (INNOVANCE D-dimer; Siemens), antithrombin (INNOVANCE; Siemens), and anti-Xa (Biophen Heparin LRT; Hyphen Biomed, Neuville-Sur-Oise, France) were measured on a Sysmex CS2100i (Sysmex Corporation, Kobe, Hyogo, Japan) hemostasis analyzer. Samples for the anti-Xa test were first diluted 2x with pooled reference plasma containing ∼100% ATIII and the anti-Xa activity was subsequently determined using specific calibration lines for UFH (aXa-UFH) (Biophen UFH Calibrator; Hyphen Biomed) or low-molecular-weight heparin (LMWH) (aXa-LMWH) (Biophen Heparine Calibrator; Hyphen Biomed). The aPTT (Cephascreen; Stago, Paris, France) was also performed on a STA-R Max2 analyzer (Stago). Thrombocyte count was determined using a Sysmex XN-9000 analyzer (Sysmex). C-reactive protein (CRP, third generation, Roche Diagnostics, Basel, Switzerland) and ferritin (Elecsys ferritin, Roche) were performed on the COBAS8000 by Roche Diagnostics.

### Statistical Analysis

The seven patients of this substudy were compared with the 81 patients of the MaastrICCht cohort. Means ± standard deviation, median (IQR) or percentages are presented and compared using independent Student's *t* -test, or chi-squared test as appropriate, with a *p* -value of 0.05 being considered statistically significant.

Associations between the different variables were made using simple linear regression and Bland–Altman analysis.

## Results

Table 1 shows that there were no differences between the seven patients and the entire MaastrICCht cohort, except that they were younger, had a relatively higher prevalence of ECMO and CRRT, and a lower Simplified Acute Physiology Score II. All included patients experienced heparin resistance.

**Table 1:** Comparison of the patient characteristics between the Maastricht Intensive Care COVID cohort (MaastrICCht) and the seven individual cases of the present subcohort

Variables	MaastrICCht–Subcohort ( *n* = 74)	Subcohort a ( *n* = 7)	*p* -Value
**General**			b
Age (y), mean (SD)	65.7 (11.0)	52.0 (17.9)	0.004
Gender (male), *n* (%)	58 (78.4)	5 (71.4)	0.677
Height (cm), mean (SD)	175.4 (8.6)	174.9 (10.4)	0.869
Weight (kg), mean (SD)	84.8 (13.5)	83.1 (9.5)	0.747
Body mass index (kg/m ^2^ ), mean (SD)	27.6 (4.3)	27.2 (2.5)	0.796
**Chronic health conditions**			c
Hypertension (yes), *n* (%)	26 (35.1)	2 (28.6)	1.000
Dyslipidemia (yes), *n* (%)	15 (20.3)	1 (14.3)	1.000
Diabetes Mellitus (yes), *n* (%)	13 (17.6)	0 (0)	0.591
Chronic kidney disease (yes), *n* (%)	1 (1.4)	1 (14.3)	0.166
Malignancy (yes), *n* (%)	6 (8.1)	0 (0)	1.000
Liver disease (yes), *n* (%)	1 (1.4)	0 (0)	1.000
Chronic lung disease (yes), *n* (%)	8 (10.8)	0 (0)	1.000
Myocardial infarction (yes), *n* (%)	3 (4.1)	0 (0)	1.000
Congestive heart failure (yes), *n* (%)	1 (1.4)	0 (0)	1.000
Peripheral vascular disease (yes), *n* (%)	2 (2.7)	1 (14.3)	0.240
CVA of TIA (yes), *n* (%)	10 (13.5)	0 (0)	0.588
Dementia (yes), *n* (%)	0 (0)	0 (0)	NA
Connective tissue disease (yes), *n* (%)	0 (0)	0 (0)	NA
Peptic ulcer disease (yes), *n* (%)	1 (1.4)	0 (0)	1.000
Immunosuppression (yes), *n* (%)	2 (2.7)	0 (0)	1.000
AIDS (yes), n (%)	0 (0)	0 (0)	NA
**Disease severity algorithms**			b
APACHE II, mean (SD)	16.0 (6.0)	15.7 (4.4)	0.916
SAPS II, mean (SD)	41.3 (13.9)	30.1 (8.5)	0.041
**Advanced therapy**			c
ECMO (yes), *n* (%)	5 (6.8)	3 (42.9)	0.019
CRRT (yes), *n* (%)	11 (14.9)	4 (57.1)	0.020
Mechanical ventilation (yes), *n* (%)	74 (100)	7 (100)	NA
Heparin therapy (yes), *n* (%)	17 (23.0)	7 (100)	<0.01
Heparin resistance (yes), *n* (%) d	10 (13.5)	7 (100)	<0.01

Abbreviations: APACHE II, Acute Physiology And Chronic Health Evaluation II; CRRT, continuous renal replacement therapy; MaastrICCht, Maastricht Intensive Care COVID cohort; NA, not applicable; SAPS II, Simplified Acute Physiology; Score II, ECMO, extracorporeal membrane oxygenation; SD, standard deviation.

a The subcohort is part of the Maastricht Intensive Care COVID cohort.

b Independent sample *t* -test for equal variances.

c Comparing groups by the Fisher's exact test.

d Defined by >35.000 U/24 hours.

**Fig. 1:**
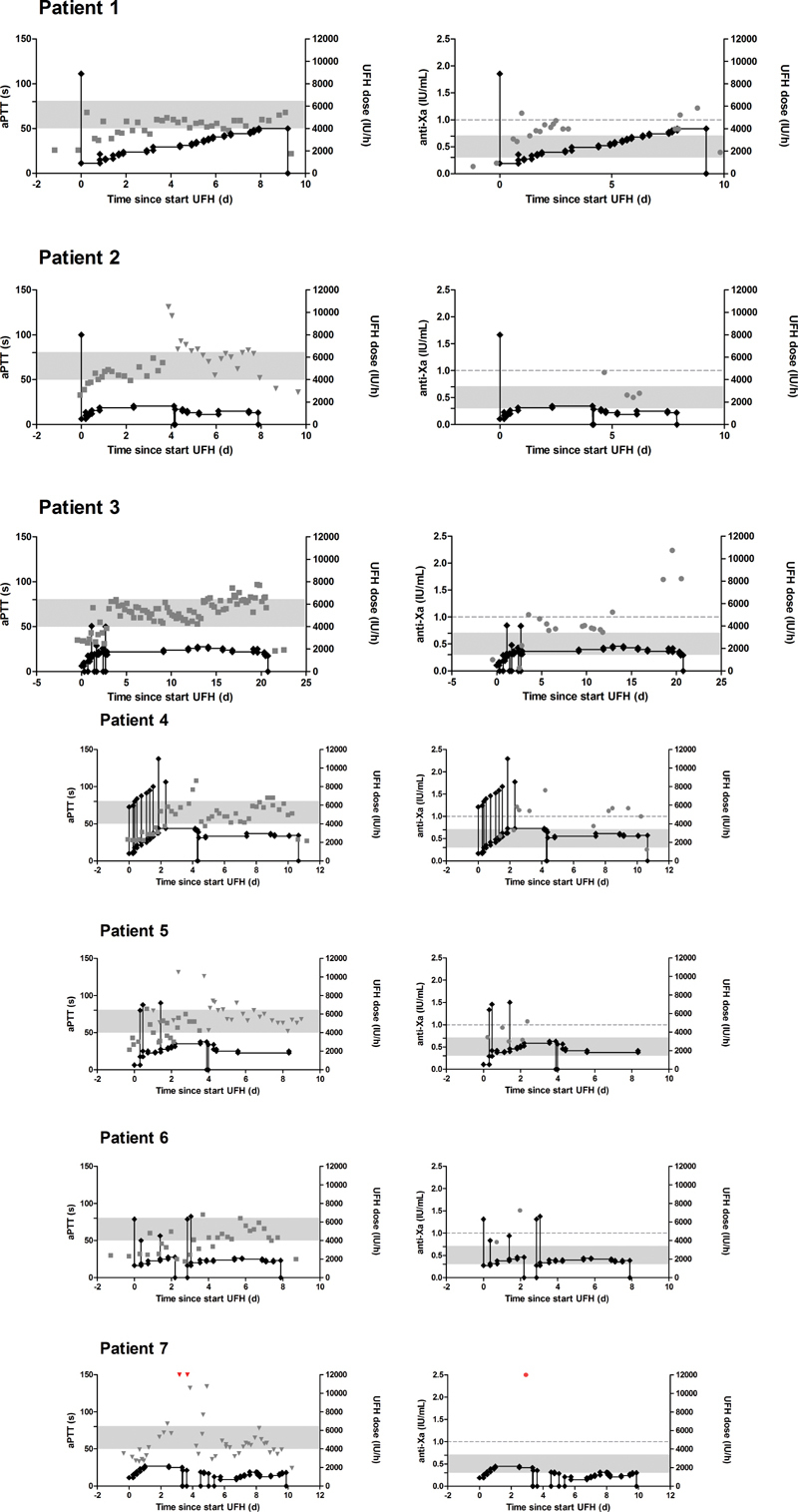
Longitudinal data of 7 patients treated with unfractionated heparin (UFH) and on extracorporeal membrane oxygenation (ECMO) or continuous venovenous hemofiltration. The left-hand panels show the activated partial thromboplastin time (aPTT) (measured with the Sysmex CS2100i (optical method),  ; or with the Stago STA-R Max 2 (mechanical method),  plotted on the left y-axis. The right-hand panels show the anti-Xa activity (  ) plotted on the left y-axis. All figures also show the actual administered UFH dose in IU/h (  ), plotted on the right y-axis. Bolus injections are in IU and are shown as vertical spikes. Shaded area indicates the heparin therapeutic range (50–80 seconds for the aPTT, 0.3–0.7 IU/mL for the anti-Xa). Dashed lines indicate the 1.0 IU/mL anti-Xa cutoff. Red symbols in patient 7 indicate aPTT values > 150 seconds and anti-Xa values > 2.5 IU/mL.

The actual heparin dose (in IU/h) and bolus injections (in IU) administered to each patient are depicted in Fig. 1 , together with the aPTT (left panels) and the anti-Xa activity (right panels). It typically takes 1 to 2 days of an increasing dose of heparin, often combined with several bolus injections, to reach the HTR of 50 to 80 seconds After reaching the HTR, patients 3, 4, and 6 then required a relatively constant dose of heparin to maintain a stable aPTT. In patient 1, the heparin dose necessary to keep the aPTT from falling, required repeated dose increases (up to a maximum of 4,000 IU/mL). By default, the aPTT was measured on the Sysmex CS2100i, which utilizes optical clot detection. Due to interferences in the optical method (so-called early-reaction errors, or biphasic waveforms, for example caused by hemolysis or turbidity), the platform used for aPTT measurement was changed to the Stago STA-R Max 2 (which utilizes mechanical clot detection) halfway during treatment in patients 2 and 5. This change in method was accompanied by an increase in the aPTT and a gradual decrease in the UFH dosage. The aPTT of patient 7 was measured on the STA-R Max 2 since the beginning of her UFH treatment. In the majority of samples, the anti-Xa activity is consistently higher than the target HTR of 0.3 to 0.7 IU/mL, but is in most cases below the therapeutic threshold of 1.0 IU/mL. Table 2 shows additional clinical characteristics for each individual patient.

**Table 2:** Individual case description: individual cases are part of the Maastricht Intensive Care COVID cohort

Variables	Case 1	Case 2	Case 3	Case 4	Case 5	Case 6	Case 7
**Demographics**							
Age (y)	49	57	66	54	48	73	17
Sex (m/f)	M	F	m	M	m	m	f
Weight (kg)	90	78	85	88	95	80	66
BMI (kg/m ^2^ )	24.9	30.5	27.8	26	30.7	26.1	24.5
**Medical history**							
	Blanco	HT with LVH gastric bypass	UCNSTEMI	Blanco	Blanco	AF	IgAN with CKD
**Therapeutic anticoagulants**							
Indication	PE	ECMO	AF	ECMO/DVT	ECMO/PE	AF/PE	PE
UFH a	Day 4–13	Day 0–8	Day 2–23	Day 0–11	Day 1–15	Day 16–24	Day 16–26
LMWH a	Day 14–17	n/a	Day 36–37	Day 11–15	n/a	Day 0–16, 24–30	Day 14–15
**Advanced therapy**							
ECMO a	n/a	Day 0–8	n/a	Day 0–11	Day 1–15	n/a	n/a
CRRT a	Day 7–15	n/a	Day 2–31	n/a	n/a	Day 16–23	Day 4–26
**Hematological complications**							
Bleeding (yes/no) b	No	Yes	No	No	No	No	No
Thrombosis (yes/no) b	No	Yes	No	No	No	No	No

Abbreviations: AF, atrial fibrillation; BMI, body mass index; CKD, chronic kidney disease; CRRT, continuous renal replacement therapy; DVT, deep venous thrombosis; ECMO, extracorporeal membrane oxygenation; HT, hypertension; ICU, intensive care unit; IgAN, IgA nephropathy; LMWH, low-molecular-weight heparin; LVH, left ventricular hypertrophy; n/a, not applicable; NSTEMI, non-ST-elevation-myocardial-infarction; PE, pulmonary embolism; UC, ulcerative colitis; UFH, unfractionated heparin.

a Period during ICU admission.

b Complications occurred during administration of UFH.

### Comparison of Different Anti-Xa and aPTT Assays

**Fig. 2:**
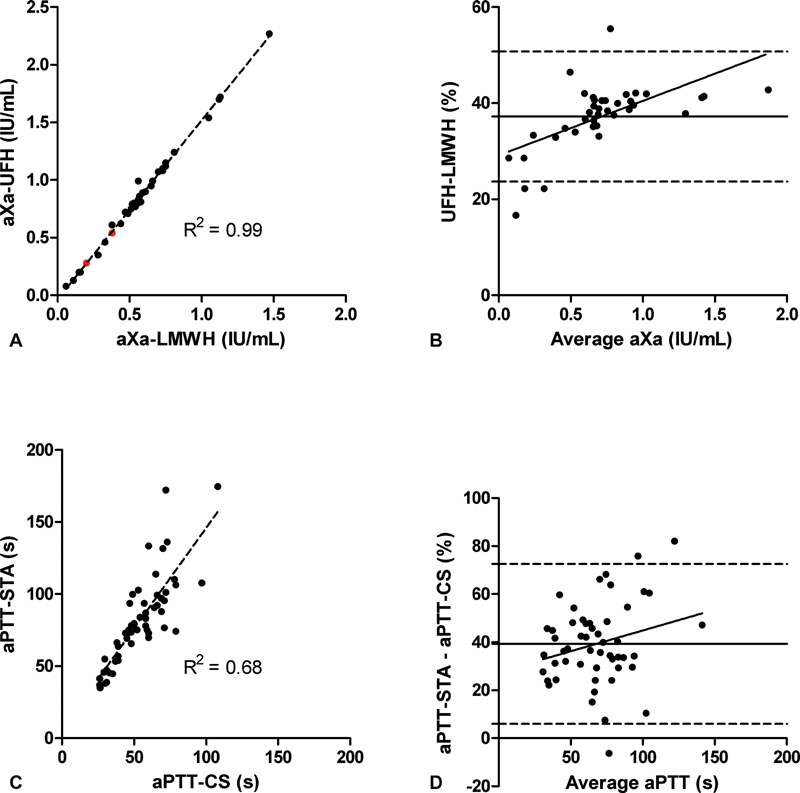
( **A** ) Correlation of the anti-Xa assay with the unfractionated heparin (UFH) and the low-molecular-weight heparin (LMWH) calibration. The two red samples are internal quality control samples for the UFH calibration. ( **B** ) Bland–Altman plot of the relative difference between the UFH and LMWH calibrations versus the average aXa activity. ( **C** ) Correlation of the activated partial thromboplastin time (aPTT) as measured with the mechanical (aPTT-STA) versus the optical (aPTT-CS) method. ( **D** ) Bland–Altman plot of the relative difference between the aPTT-STA and aPTT-CS versus the average aPTT. Bland–Altman plots show the relative bias (thick lines) and the 95% limit of agreement (dashed lines).

Next, the agreement and correlation between different anti-Xa calibrators and aPTT methods were analyzed. Fig. 2A shows the correlation between aXa-LMWH and aXa-UFH in 37 samples ( *r* ^2^ = 0.99). A factor difference of 1.55 exists between the two variables: aXa-UFH = 1.55 * aXa-LMWH. Because of the near-perfect correlation, the LMWH calibration line was used throughout this study after conversion of the values to aXa-UFH by multiplication of aXa-LMWH with 1.55. Bland–Altman analysis shows an average bias of 37.2% ( Fig. 2B ). The relative bias is significantly less below 0.4 IU/mL.

Fig. 2C shows the correlation between the optical aPTT (aPTT-CS) and the mechanical aPTT (aPTT-STA) ( *r* ^2^ = 0.68). The aPTT measured with the mechanical method is consistently higher than the aPTT measured with the optical method: aPTT-STA = 1.4 * aPTT-CS + 6. Bland–Altman analysis shows an average bias of 39.3% with an even spread across the entire measuring range ( Fig. 2D ).

### Correlation between Anti-Xa and aPTT in COVID-19 Patients

**Fig. 3:**
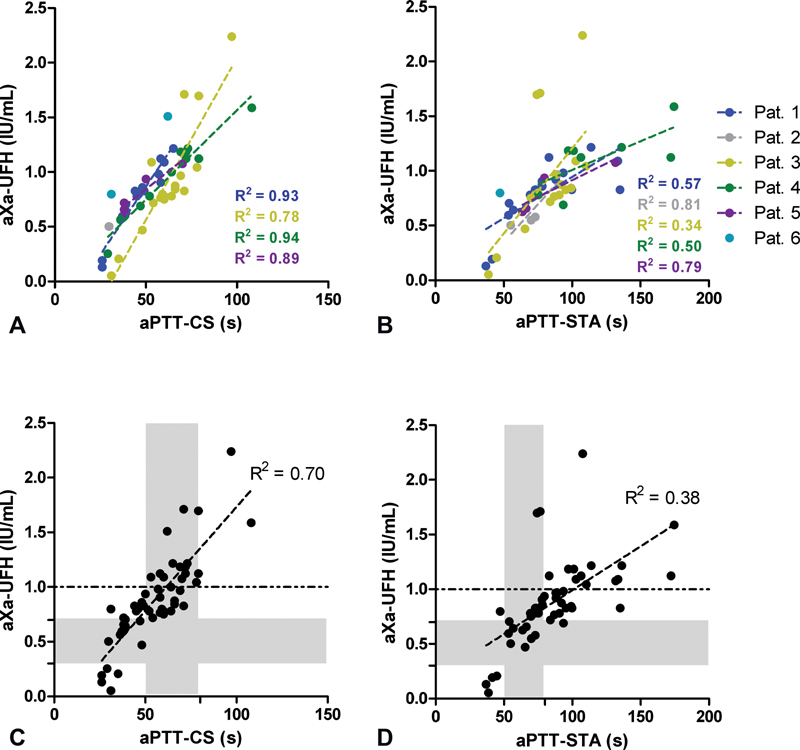
Correlation between the anti-Xa activity and the activated partial thromboplastin time (aPTT) measured on the Sysmex CS2100i (aPTT-CS) per patient ( **A** ), Correlation between the anti-Xa activity and the aPTT measured on the Stago STA-R Max 2 (aPTT-STA) per patient ( **B** ), overall correlation between the anti-Xa activity and the aPTT-CS ( **C** ), and overall correlation between the anti-Xa activity and the aPTT-STA ( **D** ). Shaded areas indicate the heparin therapeutic range of the anti-Xa assay (0.3–0.7 IU/mL) and of the aPTT (50–80 seconds for both assays). Dashed lines indicate an anti-Xa activity of 1.0 IU/mL. LMWH, low-molecular-weight heparin; UFH, unfractionated heparin.

We next compared the anti-Xa activity with the aPTT-CS and with the aPTT-STA for each patient separately. Fig. 3A shows these correlations for the anti-Xa activity and the aPTT-CS (0.78 ≤ *r* ^2^ ≤0.94). However, the slope of each line varies, suggesting that the association between anti-Xa and the aPTT varies for each individual. When comparing the aPTT-STA with the anti-Xa activity, the observed correlation (0.34 ≤ *r* ^2^ ≤ 0.81) was lower compared with the aPTT-CS, but the patient-specific slope remains ( Fig. 3B ).

Correlation without taking dependence between data of all datapoints into account was lower for both the aPTT-CS ( *r* ^2^ = 0.66, Fig. 3C ) and the aPTT-STA ( *r* ^2^ = 0.36, Fig. 3D ). No samples fall within the HTR of both aPTT (50–80s) and anti-Xa (0.3–0.7 IU/mL) when using the aPTT-CS, and only eight samples fall within the HTR of both tests when using the aPTT-STA. When an anti-Xa activity of 0.3 to 0.7 IU/mL would be used as target range, the corresponding aPTT target range would equal 24.5 to 45.6 seconds using the aPTT-CS and 13.8 to 63.8 seconds using the aPTT-STA in this population.

### Acute Phase Reactants Do Not Contribute to Differences in aPTT

Lastly, the relation of FVIII, fibrinogen, ATIII, thrombocytes, CRP, and ferritin was assessed with the aPTT-CS. Acute phase reactants were high: mean FVIII 606% (ref. 50–150%), mean fibrinogen 7.5 g/L (ref. 1.7–4.0 g/L), mean CRP 164 mg/L (<10 mg/L), mean ferritin 2072 µg/L (ref. 30–400 µg/L), and mean platelets 340 * 10^9 /L (ref. 150–350 * 10^9 / L) among all samples analyzed. ATIII was > 70% for all patients, except patient 5 (66% and 55% in 2 samples). Mean D-dimer concentration was >7210 µg/L (ref. <500 µg/L), but is likely higher because the majority of samples had D-dimer concentrations above the measurement limit. No relevant correlation was found with the aPTT and any of them (data not shown).

## Discussion

In this pilot study, we explored the usefulness of two often-used aPTT assays and a chromogenic anti-Xa assay on the monitoring of UFH in seven COVID-19 patients on CRRT or ECMO. We show that these patients required a high dosage of UFH, confirming heparin resistance (defined as >35,000 IU UFH per day) in all of them. One of our patients developed bleeding during the treatment requiring the transfusion of six packed cells over the course of several days (patient 2). Another patient developed thrombosis despite high dosage of UFH (patient 3). We next showed that it is possible to measure the anti-Xa activity of UFH using an LMWH calibration and multiplication by a factor 1.55. The thusly obtained anti-Xa activity has an excellent correlation with the aPTT as measured by the Sysmex CS2100i but has a patient-specific association, indicating that patient-specific factors contribute to the relation between anti-Xa and the aPTT. Compared with the Sysmex CS, a significantly lower correlation was found between the anti-Xa activity and the aPTT on the Stago STA-R Max2. Lastly, we found no significant relation between FVIII, fibrinogen, thrombocytes, CRP, ferritin, or ATIII activity and the aPTT.

Because of the high incidence of coagulopathy in patients with SARS-CoV-2 infections, the International Society of Thrombosis and Haemostasis (ISTH) recommends prophylactic treatment with LMWH for all patients who require hospital admission for COVID-19. 25 Despite the use of standard thrombotic prophylaxis with LMWH, the cumulative incidence of thrombotic complications in COVID-19 patients admitted to the ICU is estimated to be 26, 47, and 59% over a period of 7, 14, and 21 days, respectively. 26 27 28 White et al recently discovered that the anti-Xa recovery after spiking plasma samples with LMWH is reduced in COVID-19 plasma versus reference plasma, suggesting that a plasma factor is eliminating the LMWH. 29 Similarly, Dutt et al found that the anti-Xa activity is reduced in COVID-19 patients receiving standard prophylactic dosage of LMWH. 30 Pending the results of randomized controlled trials investigating the optimal dose of LMWH, local guidelines in the Netherlands advocate doubling of the prophylactic LMWH dose in patients on the ICU except in those with an increased bleeding tendency or severe renal insufficiency.

With regard to therapeutic UFH, the dosage can be adjusted based on the aPTT or the anti-Xa, with the aPTT being the measure of choice in many centers. 10 The anti-Xa test is believed by many to be superior to the aPTT because it achieves therapeutic anticoagulation more rapidly, maintains the values within the therapeutic window for a longer period of time, and thus requires fewer adjustments in dosage and testing. 31 32 33 The overall concordance between the aPTT and the anti-Xa test is low: 51.8% in adults, and dependent on age. 34 Interestingly, Arachchillage et al show that age is a discerning factor and that infants have a high aPTT versus adults that have a lower aPTT in relation to their anti-Xa levels, possibly explaining the patient-specific association we observed. 34 However, the anti-Xa test only measures the anti-Xa activity of heparin, while UFH also inhibits thrombin and other coagulation factors, and it ignores all other factors that may modulate its effect in vivo. An example of such an effect is the binding of FVIII and acute phase proteins to UFH, resulting in a discordancy between aPTT and anti-Xa. 35 Increased plasma concentrations of fibrinogen or FVIII may lead to aPTT shortening in 16% of patients with COVID-19. 36 We believe that in such cases, the aPTT may be a more representative test to assess the net effect of heparin on the in vivo coagulation and may therefore be superior to the anti-Xa. Uprichard et al tested this hypothesis and found that the aPTT, and not the anti-Xa, concurred with thromboelastographic and thrombin generation parameters, suggesting that apparent heparin resistance may actually be a genuine one. 37

In COVID-19 patients admitted to the ICU and treated with UFH, a severe acute phase response is present. CRP, ferritin, FVIII, fibrinogen, and D-dimer levels are extremely high as also observed in the current study. Platelet count is typically normal in these patients while lymphocyte count is reduced. 38 Patients in our subcohort may be more severely ill than those in other studies based on the severity of the acute phase response and the fact that CRRT or ECMO is required. This acute phase response is likely responsible for the high rate of heparin resistance in our patients as decreased ATIII, the most common cause of heparin resistance was not observed. In addition, high FVIII, von Willebrand Factor, and fibrinogen is associated with an increased risk of (recurrent) thromboembolism, 39 40 41 42 highlighting the need for increased anticoagulant therapy. We did not observe a significant shortening of the aPTT as a result of these acute phase reactants, but this comparison may be confounded by the fact that we used the aPTT to regulate the UFH dosage. The thrombotic nature of COVID-19 is rather controversial and may instead be due to localized immunothrombosis instead of globally increased plasma coagulability. 43 Furthermore, the normal thrombocyte count in these patients may reflect a balance between increased platelet production and consumption. In severe COVID-19 patients, platelets are activated 44 and may release content from their granules and thereby reduce the effect of heparin in vivo. Measuring of heparin cofactor 2 or platelet factor 4 may therefore be of interest in the future. Another hypothesis for the high need of UFH in these patients is heparanase activity. Heparanase is a heparan sulfate degrading enzyme, which also has affinity for UFH. 45 Heparanase activity seems to be increased during inflammatory disease including sepsis-associated lung injury or bacterial and viral infection. 46 Evidence for increased heparanase expression in COVID-19 is lacking, but based on its ability to cleave UFH it could be involved in the ethology of heparin resistance in COVID-19 and may also be a potential target for further research.

When we would have used the anti-Xa activity of 0.3 to 0.7 IU/mL instead of the aPTT ratio as a basis for the HTR in this population, it would have resulted in a HTR of 24.5 to 45.6 seconds when using the Sysmex CS2100i, or a HTR of 13.8 to 63.8 seconds when using the Stago STA-R Max2. This also suggests that using only the anti-Xa assay without the aPTT may have resulted in an underdosage of heparin in our patients. At the same time, it highlights the underlying problem of poorly standardized aPTT assays. Depending on the aPTT measuring principle in combination with the reagents used, the dosage of UFH can be significantly different between hospitals. Even though both our aPTT assays have similar reference intervals and thus an identical HTR, we see poor agreement between these two assays in this population, even leading to dosage changes in two of our patients when the aPTT was measured with a different assay. Monitoring of UFH in these two patients was switched to the aPTT-STA because a biphasic waveform was detected on the aPTT-CS. These biphasic waveforms occur frequently in critically ill patients on the ICU and are caused by the formation of a precipitate of CRP and very-low-density lipoprotein after the recalcification of plasma. 47 In COVID-19, high CRP concentrations and disseminated intravascular coagulation may cause these biphasic waveforms in optical aPTT analyzers. An interesting idea for further research would be to switch reagents between the two analyzers and to study the effect on the measured aPTT and the amount of biwaves observed.

Another point of attention is the method of anti-Xa measurement. In our laboratory, we have chosen to dilute the patient sample 1:1 with reference pool plasma, thereby supplementing ATIII. Since ATIII levels were normal in this subcohort, the difference was negligible. However, other laboratories may choose to dilute with NaCl, making this an important factor to keep in mind when interpreting laboratory parameters. All of this combined leads us to believe that it may be a mistake to forego the measuring of the aPTT in COVID-19 patients, as suggested by some colleagues. 48 The aPTT reflects the net effect of heparin on the intrinsic coagulation cascade and therefore provides more complete information than merely anti-Xa activity. No study has yet to show a clear benefit of either approach and we don't know which is better. We therefore believe that both the aPTT and the anti-Xa tests must be interpreted together, and in relation to the clinical status of the individual patients (bleeding or prothrombotic phenotype), to make the best dosage decisions. Physicians should consider to adjust the UFH dose if the HTR is not reached based on the individual patient's situation.

Our study has several limitations. Our sample size is small and underpowered for clinically relevant endpoints. The patient population is very coherent with the same underlying disease, making it possible for significant observations with smaller sample sizes but possibly decreasing generalizability. Not every aPTT is mirrored with an aXa measurement; however, the observations in this study are consistent across the sample. For the comparison of the two aPTT assays, samples that were measured on the STA-R Max2 consisted of PFP that underwent 1 freeze-thaw cycle, while the samples measured on the CS2100i were fresh samples. Furthermore, no COVID-19 patients were treated with UFH in the absence of CVVH or ECMO, nor did we have access to comparable data of COVID-negative patients on CVVH or ECMO that could be used as a control.

Neither the aPTT nor the anti-Xa assays are perfect tests. The goal of heparinization is to decrease thrombotic events without causing bleeding. Future research should be focused around thrombin generation 49 and viscoelastic testing 50 in whole blood to provide an even better understanding of all the intricacies at play in COVID-19 patients.
